# Efficacy of vortioxetine on the physical symptoms of major depressive disorder

**DOI:** 10.1177/0269881118788826

**Published:** 2018-07-26

**Authors:** Michael Cronquist Christensen, Ioana Florea, Annika Lindsten, David S Baldwin

**Affiliations:** 1 H. Lundbeck A/S, Valby, Denmark; 2 Clinical and Experimental Sciences, Faculty of Medicine, University of Southampton, UK; 3 University Department of Psychiatry and Mental Health, University of Cape Town, South Africa

**Keywords:** Vortioxetine, major depressive disorder, physical symptoms

## Abstract

**Background:**

Efficacy has been proven for vortioxetine in short-term and long-term treatment of major depressive disorder (MDD), with broad beneficial effects on emotional, physical and cognitive symptoms. Limited specific data on the effects of vortioxetine on depression-related physical symptoms have been published.

**Methods:**

A meta-analysis was carried out of five short-term multinational, double-blind, placebo-controlled studies. These studies were conducted in a total of 2105 adult MDD outpatients (18–75 years) with a major depressive episode of ⩾3 months’ duration. Only patients treated with a dose of 5 or 10 mg vortioxetine (therapeutic doses) or placebo were included in this analysis. Efficacy assessment of vortioxetine on the physical symptoms of depression included all items of the Hamilton Depression Scale (HAM-D) assessing physical symptoms, and all somatic items in the Hamilton Anxiety Scale (HAM-A). A subgroup analysis in MDD patients with coexisting anxiety symptoms (i.e. those with a HAM-A ⩾20 at baseline) was also performed.

**Results:**

A significant improvement (*p*<0.05) of vortioxetine versus placebo was observed on all HAM-D items measuring physical symptoms, except for the somatic gastrointestinal symptoms and loss of weight items. Significant effects were also observed on the HAM-A somatic items: general somatic symptoms, gastrointestinal symptoms, and autonomic symptoms. In patients with a high baseline level of anxiety, a significant effect of vortioxetine was also observed on the physical symptoms of depression.

**Conclusions:**

These analyses indicate that patients with MDD, including those with a high level of anxiety symptoms, have significant improvements in MDD-associated physical symptoms when treated with vortioxetine.

## Introduction

Major depressive disorder (MDD) is characterized by multiple debilitating symptoms, spanning emotional, physical and cognitive domains, with serious consequences for patients’ psychosocial and occupational functioning. Core symptoms of MDD include a persistent disturbance of mood and loss of interest/pleasure in most daily activities (Otte et al., 2016). Patients may also experience physical symptoms such as fatigue/low energy, sleep and appetite disturbances, muscle tension, headaches, and general symptoms of pain, and cognitive symptoms such as impaired concentration, poor memory and difficulty in making decisions (American Psychiatric Association, 2013; Singh and Gotlib, 2014).

Full functional recovery is the ultimate treatment goal for patients with MDD, but many patients do not achieve even the more limited goal of full remission of depressive symptoms (Papadimitropoulou et al., 2017): meta-analyses of controlled clinical studies indicate that only 30–50% of patients achieve remission after 6–8 weeks of antidepressant treatment (Warden et al., 2007). Patients in partial remission may still have debilitating symptoms such as insomnia, anxiety, anhedonia, apathy and memory/concentration difficulties (Fava et al., 2006; Mattingly et al., 2016; McClintock et al., 2011). The presence of residual depressive symptoms partly accounts for the prevention of full functional recovery (Judd et al., 1998), and predicts earlier relapse, recurrence and a more chronic course of illness (Judd et al., 1998, 2000; Kennedy and Paykel, 2004).

Physical symptoms are commonly observed in patients with MDD. Depressive disorder with physical (somatic) symptoms may be the most common presentation of depression in inpatient and outpatient settings (Kapfhammer, 2006). In studies reported by Hamilton (1989) and Kirmayer et al. (1993), about 80–90% of patients experienced physical symptoms, especially somatic anxiety and fatigue (Hamilton, 1989; Kirmayer et al., 1993). Further, in a meta-analysis of 14 studies of patients with depression, 65% reported pain symptoms (Bair et al., 2003). In addition, in a separate study by Fava and colleagues (2006), physical symptoms of sleepiness/sedation were reported by over 40% of patients who responded to and were continuing with long-term antidepressant treatment (Fava et al., 2006).

The common clinical focus on the psychological symptoms of depression may obscure diagnosis in patients primarily presenting with physical symptoms, emphasizing the importance of careful clinical examination to avoid missing a diagnosis of depression (Rijavec and Grubic, 2012). The presence of somatic symptoms has a detrimental effect on the course and response to treatment (Greden, 2003). Evidence also suggests that patients with somatic symptoms have a more chronic course of MDD and greater risk of comorbid anxiety disorders (Gerrits et al., 2012; Jaracz et al., 2016).

Vortioxetine has a multimodal mechanism of action (i.e., direct modulation of receptor activity and inhibition of the serotonin transporter) and has been approved for the treatment of MDD (Sanchez et al., 2015). The efficacy and safety of vortioxetine in MDD was established as part of an extensive clinical development programme, which comprised 17 short-term placebo-controlled studies, six open-label long-term extension studies and one long-term relapse–prevention study, involving more than 9700 patients and a total exposure of over 3450 patient-years (Baldwin et al., 2016a, 2016b; Florea et al., 2015; Melander et al., 2008).

Vortioxetine significantly improves depressive symptoms as measured by the Montgomery–Åsberg Depression Rating Scale (MADRS) or by the 24-item version of the Hamilton Depression Rating Scale (HAM-D) at doses between 5 and 20 mg daily (Kelliny et al., 2015). Pooled analyses of data from short-term studies reveal significantly higher response and remission rates with vortioxetine when compared with placebo (Berhan and Barker, 2014; Kelliny et al., 2015). Further, meta-analyses of effects of vortioxetine on the single items of the MADRS scale indicate its favourable effects across a broad range of depressive symptoms (Thase et al., 2016).

Favourable effects of vortioxetine extend beyond emotional symptoms. In short-term controlled studies within the 5–20 mg dose range, vortioxetine significantly improves cognitive function (executive function, processing speed and attention/concentration) as compared to placebo, as measured by the Digit Symbol Substitution Test in patients with MDD (Mahableshwarkar et al., 2015; McIntyre et al., 2016). In addition, meta-analyses of short-term (6–8 week) studies in the same dosing range indicate improved overall functioning and functional remission, as measured by the Sheehan Disability Scale in adult MDD patients (Boulenger et al., 2014; Florea et al., 2017; Wang et al., 2015), and significant and clinically meaningful improvements in health-related quality of life (Boulenger et al., 2014; Florea et al., 2015).

So far, only limited data have been published specifically addressing the effects of vortioxetine on depression-related physical symptoms. We therefore undertook post hoc analyses of data from five short-term, placebo-controlled studies of vortioxetine in patients with MDD. We chose these studies because both the 24-item HAM-D (Hamilton, 1960; Riskind et al., 1987) and the Hamilton Anxiety Rating Scale (HAM-A) (Hamilton, 1959a) were employed; these scales cover a broad range of physical symptoms, permitting a more detailed assessment of potential effects within this domain.

## Materials and methods

*Clinical studies*: All short-term studies where efficacy of vortioxetine on both the HAM-D and HAM-A were investigated in a comparable adult MDD population were included in this analysis. This comprised five short-term (6- or 8-week duration), randomized, double-blind, placebo-controlled, multi-centre studies evaluating the efficacy of vortioxetine versus placebo in adults with MDD (Table 1). Study NCT00735709 (Henigsberg et al., 2012) investigated fixed doses of 1, 5 and 10 mg/day vortioxetine. Study NCT00839423 (Alvarez et al., 2012) investigated fixed doses of 5 and 10 mg/day vortioxetine. Study NCT00635219 (Baldwin et al., 2012b) investigated fixed doses of 2.5, 5 and 10 mg/day vortioxetine, study NCT00672958 (Jain et al., 2013) investigated a fixed dose of 5 mg/day vortioxetine and study NCT00672620 (Mahableshwarkar et al., 2013) investigated fixed doses of 2.5 and 5 mg/day vortioxetine. From these studies, only patients treated with a dose within the therapeutic dose range (i.e. 5 or 10 mg/day) or placebo were considered for this analysis. All studies employed the MADRS, the 24-item HAM-D and the HAM-A as efficacy endpoints. The study population was defined as adults (aged 18‒75 years) with a primary diagnosis of MDD according to DSM IV-TR criteria, a current major depressive episode (MDE) of ⩾3 months’ duration (confirmed using the Mini International Neuropsychiatric Interview (Sheehan et al., 1998)) and a Montgomery–Åsberg Depression Rating Scale (MADRS) total score of ⩾ 22, 26 or 30 at screening and baseline visits (Alvarez et al., 2012; Baldwin et al., 2012b; Henigsberg et al., 2012; Jain et al., 2013; Mahableshwarkar et al., 2013).

**Table 1.** Summary characteristics of the five short-term, placebo-controlled studies of vortioxetine in patients with MDD included in the meta-analyses.

NCT identifier	Treatment period (weeks)	Dose(s) of vortioxetine tested (mg/day)^a^	Key inclusion criteria for MDD	Literature citation
**NCT00839423**	6	5 or 10	Between 18 and 65 years MADRS total score ⩾30	**Alvarez et al. 2012**
**NCT00635219**	8	2.5, 5 or 10	Between 18 and 75 years MADRS total score ⩾26	**Baldwin et al. 2012b**
**NCT00735709**	8	1,5 or 10	Between 18 and 75 years MADRS total score ⩾26	**Henigsberg et al. 2012**
**NCT00672958**	6	5	Between 18 and 75 years MADRS total score ⩾30	**Jain et al. 2013**
**NCT00672620**	8	2.5 or 5	Between 18 and 75 years MADRS total score ⩾22	**Mahableshwarkar et al. 2013**

a For the post hoc meta-analyses, only the 5 and 10 mg doses, approved as per current prescribing information, were included.

Detailed descriptions of the five clinical trial designs, methods and primary efficacy analyses have been published (Alvarez et al., 2012; Baldwin et al., 2012b; Henigsberg et al., 2012; Jain et al., 2013; Mahableshwarkar et al., 2013). All trials were conducted according to the principles of Good Clinical Practice (International Conference on Harmonization, 1996) the Declaration of Helsinki (World Medical Association, 1964 and 2008), and adhered to the requirements of all applicable local or regional regulations.

The meta-analysis did not include the long-term open-label studies, nor the dedicated study in elderly patients with MDD, or the study conducted for regulatory submission in Japan. The open-label studies (Alam et al., 2014; Baldwin et al., 2012a; Florea et al., 2012; Jacobsen et al., 2015a) were excluded as these by definition do not have a comparator and thus prevent establising efficacy. The dedicated elderly study (Katona et al., 2012) and the Japanese study (Inoue et al., 2018) were excluded as they did not include comparable study populations to the five global studies conducted in an adult MDD population; thus preventing a pooled analysis.

*Clinical assessments*: Complete details of all study assessments in the five studies are provided in Alvarez et al. (2012), Baldwin et al. (2012b), Henigsberg et al. (2012), Jain et al. (2013) and Mahableshwarkar et al. (2013). This analysis is based on HAM-D items assessing the physical symptoms of depression, namely insomnia (early (item 4), middle (item 5) and late (item 6)), anxiety somatic (item 11), somatic symptoms gastrointestinal (item 12), somatic symptoms general (include both muscular pain, headache and lack of energy) (item 13), genital symptoms (include both loss of libido and menstrual disturbances) (item 14) and loss of weight (item 16) (Fava, 2003; Hamilton, 1960; Hung et al., 2006), as well as the physical symptoms measured by the HAM-A, namely items of general somatic symptoms (muscular pain) (item 7), general somatic symptoms (sensory) (item 8), cardiovascular symptoms (item 9), respiratory symptoms (item 10), gastrointestinal symptoms (item 11), genitourinary symptoms (item 12) and autonomic symptoms (item 13) (Hamilton, 1959b).

*Statistical analysis*: To investigate the efficacy of vortioxetine on the physical symptoms of depression, a meta-analysis was performed, including data from all five studies. The statistical analyses were based on the full analysis set (FAS), as defined in each study separately. All statistical tests were two-sided. Nominal *p*-values less than 5% were considered statistically significant. Changes from baseline in HAM-D and HAM-A single items were, for each study and item separately, analyzed using a mixed model for repeated measurements (MMRM) approach, including treatment and site as factors and baseline value as covariate, with treatment-by-week and baseline-by-week interactions, and using an unstructured variance–covariance matrix. The MMRM analyses included all dose groups included in each study, but results were re-analyzed to align the model across studies before applying the meta-analysis. Standard random effects meta-analyses were carried out using the HAM-D and HAM-A results from the studies, and standardized mean differences to placebo were derived. The standardized estimates (SES) were obtained by applying a Cohen’s D approach in the MMRM setting, with the relevant denominator being derived directly from the MMRM standard error to obtain the same *p*-values for the SES as for the original estimates.

The same meta-analysis was repeated but only including the data from the three studies – NCT00839423, NCT00635219 and NCT00735709 – that separated from placebo on the primary endpoint using the same analysis applied in our research, namely MMRM (Alvarez et al., 2012; Baldwin et al., 2012b; Henigsberg et al., 2012). As the 10 mg dose was only investigated in these three studies, the results for the 10 mg group are identical to those in the meta-analysis considering all five studies. In addition, patients with a significant level of anxiety symptoms (i.e. those with a HAM-A ⩾20 at baseline) were analyzed as a subgroup. MDD patients with coexisting anxiety symptoms are not only common but typically also more difficult to treat than MDD patients without prominent anxiety, hence form a clinically relevant subgroup for this analysis (Hirschfeld, 2001).

The trial was registered at ClinicalTrials.gov identifier: NCT00839423, NCT00635219, NCT00735709, NCT00672958, NCT00672620.

## Results

*Baseline characteristics*: Across studies, a total of 2105 patients were randomized to double-blind treatment with placebo (*n* = 850), vortioxetine 5 mg (*n* = 861) or vortioxetine 10 mg (*n* = 394). Of these, 2089 received study medication and 1729 completed the 6/8-week treatment period. Premature discontinuation rates were 17.7%, 16.6% and 17.7% in the placebo, vortioxetine 5 mg and vortioxetine 10 mg groups, respectively, across studies.

Demographic and baseline clinical characteristics of the study population in all five studies are summarized in Table 2. Baseline demographic and clinical characteristics were similar across treatment groups. In the five studies patients had a mean age of approximately 44 years, all groups comprised a greater proportion of women than men and patients were predominantly Caucasian. The mean baseline MADRS total score and HAM-A total score were approximately 32 and 21, respectively, across all treatment groups in all five studies indicating a patient population with moderate to severe MDD and a significant level of anxiety.

**Table 2.** Demographic and baseline characteristics (all randomized patients).

Variable	**All studies** (NCT00839423, NCT00635219, NCT00735709, NCT00672958, NCT00672620)			
	Placebo (*n*=850)	Vortioxetine 5 mg (*n*=861)	Vortioxetine 10 mg (*n*=394)	Total (*n*=2105)
**Age, years**				
Mean (SD)	43.3 (12.6)	43.9 (13.0)	44.9 (12.9)	43.8 (12.8)
Range	18-75	18-75	18-75	18-75
**Gender,** *n* (%)				
Male	333 (39.2)	306 (35.5)	141 (35.8)	780 (37.1)
Female	517 (60.8)	555 (64.5)	253 (64.2)	1325 (62.9)
**Race** (grouped) *n* (%)				
Caucasian	673 (79.2)	662 (76.9)	321 (81.5)	1656 (78.7)
Black	112 (13.2)	125 (14.5)	6 (1.5)	243 (11.5)
Asian	61 (7.2)	69 (8.0)	64 (16.2)	194 (9.2)
American	3 (0.4)	1 (0.1)	0	4 (0.2)
	1 (0.1)	2 (0.2)	3 (0.8)	6 (0.3)
**BMI, kg/m** ^2^				
Mean (SD)	28.3 (6.9)	28.4 (7.5)	25.5 (4.8)	27.8 (6.9)
**No. previous MDEs**				
Mean (SD)	2.3 (2.6)	2.4 (3.1)	1.7 (2.1)	2.2 (2.7)
Range	0–23	0–45	0–20	0–45
**Duration of current MDE, weeks**				
Mean (SD)	35.5 (57.4)	40.8 (92.0)	30.6 (64.3)	36.8 (74.7)
**MADRS total score**				
Mean (SD)	32.3 (4.0)	32.6 (4.1)	32.3 (3.7)	32.4 (4.0)
**CGI-S score**				
Mean (SD)	4.8 (0.7)	4.8 (0.7)	4.9 (0.7)	4.8 (0.7)
**HAM-D,** mean (SD)				
Item 4: Insomnia Early^1^	1.5 (0.7)	1.5 (0.8)	1.5 (0.7)	1.5 (0.8)
Item 5: Insomnia Middle^2^	1.5 (0.7)	1.5 (0.7)	1.5 (0.7)	1.5 (0.7)
Item 6: Insomnia Late^3^	1.4 (0.8)	1.3 (0.8)	1.3 (0.7)	1.3 (0.8)
Item 11: Anxiety Somatic^4^	1.6 (0.8)	1.6 (0.8)	1.7 (0.7)	1.6 (0.8)
Item 12: Somatic Symptoms: Gastrointestinal^5^	0.8 (0.6)	0.8 (0.6)	0.8 (0.6)	0.8 (0.6)
Item 13: Somatic Symptoms: General^6^	1.6 (0.6)	1.6 (0.6)	1.5 (0.6)	1.6 (0.6)
Item 14: Genital Symptoms^7^	1.3 (0.8)	1.3 (0.8)	1.2 (0.8)	1.3 (0.8)
Item 16: Loss of Weight^8^	0.3 (0.7)	0.3 (0.7)	0.5 (0.7)	0.4 (0.7)
Total score (24 items)	31.0 (5.5)	31.5 (5.6)	31.1 (5.5)	31.2 (5.5)
**HAM-A,** Mean (SD)				
Item 7: Somatic Muscular^9^	1.2 (0.9)	1.2 (0.9)	1.3 (0.9)	1.3 (0.9)
Item 8: Somatic Sensory^10^	0.8 (0.9)	0.8 (0.9)	1.1 (0.9)	0.9 (0.9)
Item 9: Cardiovascular^11^	0.8 (0.9)	0.7 (0.9)	1.1 (0.9)	0.8 (0.9)
Item 10: Respiratory^12^	0.7 (0.8)	0.7 (0.8)	0.9 (0.9)	0.7 (0.8)
Item 11: Gastrointestinal^13^	1.0 (0.9)	1.0 (0.9)	1.3 (0.9)	1.1 (0.9)
Item 12: Genitourinary^14^	1.5 (1.1)	1.5 (1.0)	1.5 (1.0)	1.5 (1.0)
Item 13: Autonomic^15^	1.2 (0.9)	1.1 (0.9)	1.3 (0.9)	1.2 (0.9)
Total score	20.4 (6.3)	20.3 (6.1)	22.3 (6.6)	20.7 (6.3)

1 Item 4: Insomnia Early = complains of occasional difficulty falling asleep, i.e. more than ½ hour, or complains of nightly difficulty falling asleep;

2 Item 5: Insomnia Middle = patient complains of being restless and disturbed during the night or waking during the night;

3 Item 6: Insomnia Late = waking in early hours of the morning but goes back to sleep or unable to fall asleep again if getting out of bed;

4 Item 11: Anxiety Somatic = physiological concomitants of anxiety, such as gastrointestinal – dry mouth, wind, indigestion, diarrhoea, cramps, belching, cardiovascular (palpitations, headache), respiratory (hyperventilation, sighing, urinary frequency, sweating);

5 Item 12: Somatic Symptoms – Gastrointestinal = loss of appetite but eating without staff encouragement, heavy feelings in abdomen, difficulty eating without staff urging, requests or requires laxatives or medication for bowels or medication for GI symptoms;

6 Item 13: Somatic Symptoms – General = heaviness in limbs, back or head, backaches, headache, muscle aches, loss of energy and fatigability;

7 Item 14: Genital Symptoms = symptoms such as loss of libido or menstrual disturbances;

8 Item 16: Loss of Weight = probable weight loss associated with present illness or definite (according to patient) weight loss.

HAM-A: ^9^Item 7: Somatic Muscular = includes weakness, stiffness, soreness merging into real pain, which is more or less diffusely localized in the muscles;

10 Item 8: Somatic Sensory = includes increased fatigability and weakness merging into real functional disturbances of the senses;

11 Item 9: Cardiovascular = includes tachycardia, palpitations, oppression, chest pain, throbbing in the blood vessels and feelings of fainting;

12 Item 10: Respiratory = includes feelings of constriction or contraction in throat or chest, dyspnoea merging into choking sensations and sighing respiration;

13 Item 11: Gastrointestinal = includes difficulties in swallowing, “sinking” sensation of the stomach, dyspepsia (heartburn or burning sensations in the stomach, abdominal pains related to meals, fullness, nausea and vomiting), abdominal rumbling and diarrhoea;

14 Item 12: Genitourinary = includes non-organic or psychic symptoms such as frequent or more pressing passing of urine, menstrual irregularities, anorgasmia, dyspareunia, premature ejaculation, loss of erection;

15 Item 13: Autonomic = includes dryness of mouth, blushing or pallor, sweating and dizziness.

**Figure 1.**
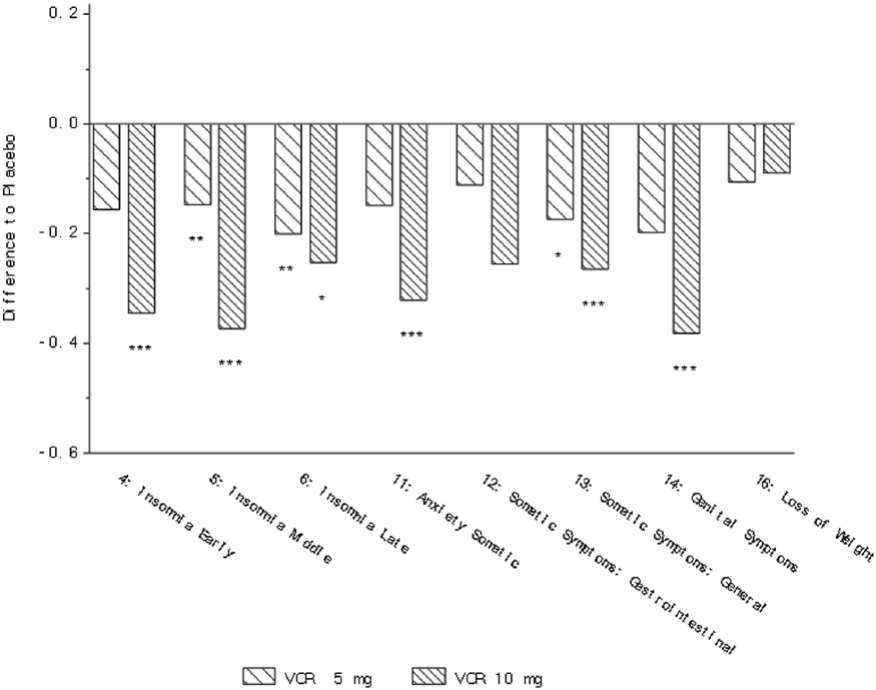
Change from baseline in HAM-D single items at week 6/8 (FAS, MMRM, SES) – all five studies (Alvarez et al., 2012; Baldwin et al., 2012b; Henigsberg et al., 2012; Jain et al., 2013; Mahableshwarkar et al., 2013). *p<0.05; **p<0.01; ***p<0.001. Definition of each item provided in Table 2.

*Clinical outcomes*: In the analysis of the five studies (NCT00839423, NCT00635219, NCT00735709, NCT00672958 and NCT00672620), a significant effect of vortioxetine versus placebo was observed in change from baseline on the HAM-D items of early insomnia (10 mg), middle and late insomnia (5 and 10 mg), anxiety somatic (10 mg), somatic symptoms general (5 and 10 mg) and genital symptoms (10 mg) (Table 3, Figure 1). For physical symptoms as measured by the HAM-A scale, a significant effect of vortioxetine versus placebo was observed on the somatic muscular item (5 mg), genitourinary item (5 and 10 mg) and autonomic item (10 mg) (Table 4). In the subgroup of MDD patients with a high baseline level of anxiety, a significant effect of vortioxetine versus placebo was observed on the HAM-D scale for insomnia early and middle (5 and 10 mg), insomnia late (5 mg), anxiety somatic (5 and 10 mg), somatic symptoms gastrointestinal (5 mg), somatic symptoms general (5 and 10 mg) and genital symptoms (5 and 10 mg) (Table 5). For the HAM-A scale a significant effect was observed on the somatic muscular (5 mg) and genitourinary items (5 and 10 mg) in patients with coexisting anxiety (Table 5).

**Table 3.** Meta-analysis of change from baseline in HAM-D single items at week 6/8 (FAS, MMRM, SES).

**Item**	**Treatment** ^a^	**All studies analysis** (NCT00839423, NCT00635219, NCT00735709, NCT00672958, NCT00672620)					**Three studies analysis** (NCT00839423, NCT00635219, NCT00735709)				
		***N***	**∆ Placebo**	**SE**	***p*-value**	**Heterogeneity** *p*-value	***N***	**∆ Placebo**	**SE**	***p*-value**	**Heterogeneity** *p*-value
**4: Insomnia Early** ^b^	Placebo	691	·	·	·		338	·	·	·	
	VOR 5 mg	714	−0.16	0.09	0.086	0.025	350	−0.28	0.08	<0.001	0.358
	VOR 10 mg	324	−0.35	0.08	<0.001	0.386	324	−0.35	0.08	<0.001	0.386
**5: Insomnia Middle** ^b^	Placebo	691	·	·	·		338	·	·	·	
	VOR 5 mg	714	−0.15	0.05	0.006	0.576	350	−0.23	0.08	0.003	0.985
	VOR 10 mg	324	−0.37	0.08	<0.001	0.815	324	−0.37	0.08	<0.001	0.815
**6: Insomnia Late** ^b^	Placebo	691					338				
	VOR 5 mg	714	−0.20	0.07	0.002	0.214	350	−0.29	0.09	<0.001	0.269
	VOR 10 mg	324	−0.25	0.12	0.038	0.088	324	−0.25	0.12	0.038	0.088
**11: Anxiety Somatic** ^b^	Placebo	691	·	·	·		338	·	·	·	
	VOR 5 mg	714	−0.15	0.08	0.059	0.078	350	−0.28	0.08	<0.001	0.678
	VOR 10 mg	324	−0.32	0.08	<0.001	0.356	324	−0.32	0.08	<0.001	0.356
**12: Somatic Symptoms: Gastrointestinal** ^b^	Placebo	691	·	·	·		338	·	·	·	
	VOR 5 mg	714	−0.11	0.07	0.091	0.205	350	−0.23	0.08	0.003	0.908
	VOR 10 mg	324	−0.26	0.18	0.153	0.005	324	−0.26	0.18	0.153	0.005
**13: Somatic Symptoms: General** ^b^	Placebo	691	·	·	·		338	·	·	·	
	VOR 5 mg	714	−0.17	0.07	0.013	0.154	350	−0.28	0.08	<0.001	0.411
	VOR 10 mg	324	−0.27	0.08	<0.001	0.408	324	−0.27	0.08	<0.001	0.408
**14: Genital Symptoms** ^b^	Placebo	691	·	·	·		338	·	·	·	
	VOR 5 mg	714	−0.20	0.10	0.052	0.007	350	−0.37	0.08	<0.001	0.853
	VOR 10 mg	324	−0.38	0.08	<0.001	0.803	324	−0.38	0.08	<0.001	0.803
**16: Loss of Weight** ^b^	Placebo	691	·	·	·		338	·	·	·	
	VOR 5 mg	714	−0.11	0.08	0.208	0.050	350	−0.04	0.08	0.620	0.941
	VOR 10 mg	324	−0.09	0.08	0.249	0.830	324	−0.09	0.08	0.249	0.830
**HAM-D24 total score**	Placebo	691					338				
	VOR 5 mg	714	−0.32	0.11	0.003	0.004	350	−0.47	0.09	<0.001	0.230
	VOR 10 mg	324	−0.58	0.15	<0.001	0.026	324	−0.58	0.15	<0.001	0.026

a The 10 mg vortioxetine (VOR) dose was tested only in the three positive studies; thus, the data in the 10 mg dose rows for the ‘All studies’ and the ‘Three studies’ analyses are identical.

b Definition of item provided in Table 2.

**Table 4.** Meta-analysis of change from baseline in HAM-A single items at week 6/8 (FAS, MMRM, SES).

**Item**	**Treatment** ^a^	**All studies analysis** (NCT00839423, NCT00635219, NCT00735709, NCT00672958, NCT00672620)					**Three studies analysis** (NCT00839423, NCT00635219, NCT00735709)				
		***N***	**∆ Placebo**	**SE**	***p*-value**	**Heterogeneity** *p*-value	***N***	**∆ Placebo**	**SE**	***p*-value**	**Heterogeneity** ***p*-value**
**7: Somatic Muscular** ^b^	Placebo	695	·	·	·		335	·			
	VOR 5 mg	711	−0.15	0.06	0.021	0.239	344	−0.21	0.08	0.005	0.740
	VOR 10 mg	319	−0.15	0.08	0.055	0.837	319	−0.15	0.08	0.055	0.837
**8: Somatic Sensory** ^b^	Placebo	695	·	·	·		335	·			
	VOR 5 mg	711	−0.10	0.05	0.052	0.866	344	−0.15	0.08	0.050	0.765
	VOR 10 mg	319	−0.18	0.11	0.126	0.121	319	−0.18	0.11	0.126	0.121
**9: Cardiovascular** ^b^	Placebo	695	·	·	·		335	·			
	VOR 5 mg	711	−0.04	0.12	0.722	0.001	344	−0.18	0.11	0.097	0.140
	VOR 10 mg	319	−0.17	0.13	0.182	0.073	319	−0.17	0.13	0.182	0.073
**10: Respiratory** ^b^	Placebo	694	·	·	·		335	·			
	VOR 5 mg	711	−0.16	0.09	0.074	0.035	344	−0.24	0.11	0.030	0.133
	VOR 10 mg	319	−0.19	0.11	0.084	0.131	319	−0.19	0.11	0.084	0.131
**11: Gastrointestinal** ^b^	Placebo	695	·	·	·		335	·			
	VOR 5 mg	711	−0.10	0.05	0.060	0.540	344	−0.20	0.08	0.011	0.990
	VOR 10 mg	319	−0.22	0.12	0.069	0.089	319	−0.22	0.12	0.069	0.089
**12: Genitourinary** ^b^	Placebo	695	·	·	·		335	·			
	VOR 5 mg	711	−0.15	0.06	0.020	0.251	344	−0.24	·0.08	0.002	0.457
	VOR 10 mg	319	−0.28	0.08	<.001	0.553	319	−0.28	0.08	<0.001	0.553
**13: Autonomic** ^b^	Placebo	695	·	·	·		335	·			
	VOR 5 mg	711	−0.01	0.07	0.897	0.206	344	−0.12	0.08	0.123	0.536
	VOR 10 mg	319	−0.27	0.12	0.025	0.103	319	−0.27	0.12	0.025	0.103
**HAM-A total score**	Placebo	695					335				
	VOR 5 mg	711	−0.25	0.10	0.012	0.012	344	−0.39	0.08	<0.001	0.370
	VOR 10 mg	319	−0.47	0.12	<.001	0.119	319	−0.47	0.12	<0.001	0.119

a The 10 mg vortioxetine (VOR) dose was tested only in the three positive studies; thus, the data in the 10 mg dose rows for the ‘All studies’ and the ‘Three studies’ analyses are identical.

b Definition of item provided in Table 2.

**Table 5.** Meta-analysis of change from baseline in HAM-D and HAM-A single items at week 6/8 in MDD patients with a high level of baseline anxiety (HAM-A total score ⩾20) (FAS, MMRM, SES).

**Item**	**Treatment** ^a^	**All studies analysis** (NCT00839423, NCT00635219, NCT00735709, NCT00672958, NCT00672620)					**Three studies analysis** (NCT00839423, NCT00635219, NCT00735709)				
		***N***	**∆ Placebo**	**SE**	***p*-value**	**Heterogeneity** *p*-value	***N***	**∆ Placebo**	**SE**	***p*-value**	**Heterogeneity** *p*-value
**HAM-D**											
**4: Insomnia Early** ^b^	Placebo	325	·	·	·		196	·	·	·	
	VOR 5 mg	315	−0.20	0.09	0.022	0.303	178	−0.29	0.13	0.029	0.189
	VOR 10 mg	183	−0.23	0.10	0.025	0.643	183	−0.23	0.10	0.025	0.643
**5: Insomnia Middle** ^b^	Placebo	325	·	·	·		196	·	·	·	
	VOR 5 mg	315	−0.19	0.08	0.018	0.772	178	−0.24	0.10	0.022	0.614
	VOR 10 mg	183	−0.39	0.10	<0.001	0.652	183	−0.39	0.10	<0.001	0.652
**6: Insomnia Late** ^b^	Placebo	325		·	·		196		·	·	
	VOR 5 mg	315	·–0.26	0.10	0.009	0.195	178	−0.29	0.18	0.098	0.058
	VOR 10 mg	183	−0.19	0.19	0.316	0.032	183	−0.19	0.19	0.316	0.032
**11: Anxiety Somatic** ^b^	Placebo	325	·	·	·		196	·	·	·	
	VOR 5 mg	315	−0.21	0.10	0.039	0.165	178	−0.32	0.13	0.012	0.218
	VOR 10 mg	183	−0.26	0.10	0.012	0.869	183	−0.26	0.10	0.012	0.869
**12: Somatic Symptoms: Gastrointestinal** ^b^	Placebo	325	·	·	·		196	·	·	·	
	VOR 5 mg	315	−0.17	0.08	0.032	0.555	178	−0.17	0.13	0.168	0.230
	VOR 10 mg	183	−0.21	0.20	0.315	0.021	183	−0.21	0.20	0.315	0.021
**13: Somatic Symptoms: General** ^b^	Placebo	325	·	·	·		196	·	·	·	
	VOR 5 mg	315	−0.27	0.08	0.002	0.341	178	−0.33	0.10	0.001	0.591
	VOR 10 mg	183	−0.28	0.10	0.006	0.617	183	−0.28	0.10	0.006	0.617
**14: Genital Symptoms** ^b^	Placebo	325	·	·	·		196	·	·	·	
	VOR 5 mg	315	−0.21	0.08	0.007	0.692	178	−0.30	0.10	0.004	0.776
	VOR 10 mg	183	−0.38	0.10	<0.001	0.929	183	−0.38	0.10	<0.001	0.929
**16: Loss of Weight** ^b^	Placebo	325	·	·	·		196	·	·	·	
	VOR 5 mg	315	−0.11	0.08	0.148	0.434	178	−0.07	0.11	0.523	0.316
	VOR 10 mg	183	−0.13	0.10	0.190	0.878	183	−0.13	0.10	0.190	0.878
**HAM-D24 total score**	Placebo	325					196				
	VOR 5 mg	315	−0.37	0.11	0.001	0.088	178	−0.49	0.18	0.005	0.060
	VOR 10 mg	183	−0.52	0.18	0.004	0.044	183	−0.52	0.18	0.004	0.044
**Item**	**Treatment**	***N***	∆ **Placebo**	**SE**	***p*-value**	**Heterogeneity** ***p*-value**	***N***	∆ **Placebo**	**SE**	***p*-value**	**Heterogeneity** ***p*-value**
**HAM-A**											
**7: Somatic Muscular** ^b^	Placebo	327	·	·	·		196	·	·	·	
	VOR 5 mg	316	−0.20	0.08	0.011	0.784	178	−0.19	0.10	0.066	0.641
	VOR 10 mg	183	−0.06	0.10	0.541	0.897	183	−0.06	0.10	0.541	0.897
**8: Somatic Sensory** ^b^	Placebo	327	·	·	·		196	·	·	·	
	VOR 5 mg	316	−0.11	0.08	0.162	0.655	178	−0.15	0.10	0.142	0.472
	VOR 10 mg	183	−0.11	0.15	0.457	0.125	183	−0.11	0.15	0.457	0.125
**9: Cardiovascular** ^b^	Placebo	327	·	·	·		196				
	VOR 5 mg	316	−0.10	0.14	0.476	0.017	178	−0.23	0.14	0.088	0.182
	VOR 10 mg	183	−0.17	0.14	0.237	0.154	183	−0.17	0.14	0.237	0.154
**10: Respiratory** ^b^	Placebo	326	·	·	·		196	·	·	·	
	VOR 5 mg	316	−0.14	0.11	0.187	0.133	178	−0.23	0.15	0.126	0.135
	VOR 10 mg	183	−0.24	0.14	0.084	0.173	183	−0.24	0.14	0.084	0.173
**11: Gastrointestinal** ^b^	Placebo	327	·	·	·		196	·	·	·	
	VOR 5 mg	316	−0.12	0.08	0.120	0.441	178	−0.17	0.10	0.096	0.633
	VOR 10 mg	183	−0.27	0.15	0.072	0.129	183	−0.27	0.15	0.072	0.129
**12: Genitourinary** ^b^	Placebo	327	·	·	·		196	·	·	·	
	VOR 5 mg	316	−0.17	0.08	0.029	0.853	178	−0.18	0.10	0.090	0.543
	VOR 10 mg	183	−0.25	0.12	0.035	0.273	183	−0.25	0.12	0.035	0.273
**13: Autonomic** ^b^	Placebo	327	·	·	·		196	·	·	·	
	VOR 5 mg	316	−0.06	0.09	0.536	0.246	178	−0.16	0.14	0.244	0.179
	VOR 10 mg	183	−0.28	0.16	0.077	0.098	183	−0.28	0.16	0.077	0.098
											
**HAM-A total score**	Placebo	327		0.			196				
	VOR 5 mg	316	−0.28	12	0.021	0.063	178	−0.42	0.17	0.013	0.076
	VOR 10 mg	183	−0.44	0.18	0.017	0.045	183	−0.44	0.18	0.017	0.045

a The 10 mg vortioxetine (VOR) dose was tested only in the three positive studies; thus, the data in the 10 mg dose rows for the ‘All studies’ and the ‘Three studies’ analyses are identical.

b Definition of item provided in Table 1.

**Figure 2.**
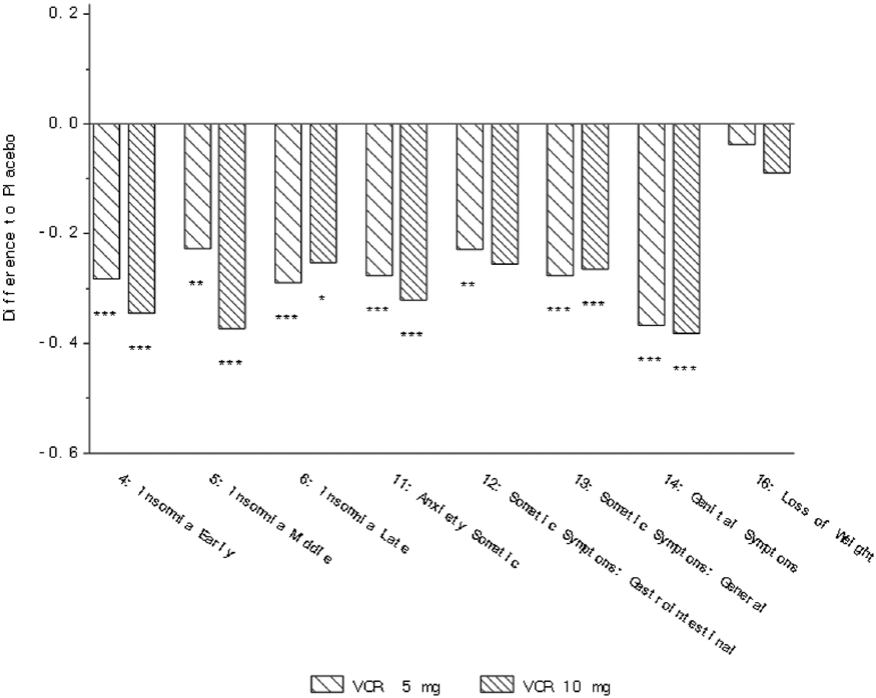
Change from baseline in HAM-D single items at week 6/8 (FAS, MMRM, SES) – three studies (Alvarez et al., 2012; Baldwin et al., 2012b; Henigsberg et al., 2012). *p<0.05; **p<0.01; ***p<0.001. Definition of each item provided in Table 2.

**Figure 3.**
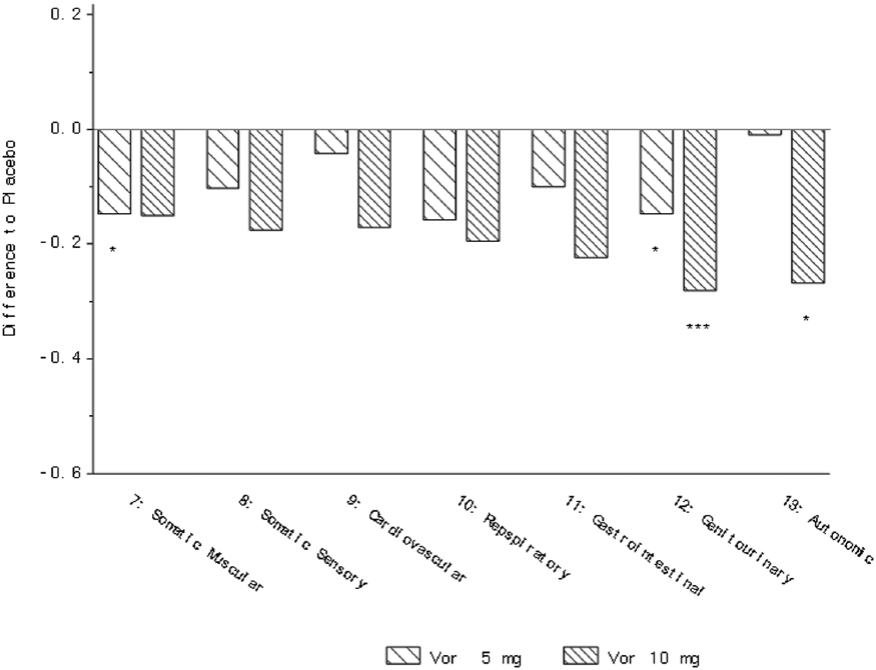
Change from baseline in HAM-A single items at week 6/8 (FAS, MMRM, SES) – five studies (Alvarez et al., 2012; Baldwin et al., 2012b; Henigsberg et al., 2012; Jain et al., 2013; Mahableshwarkar et al., 2013). *p<0.05; **p<0.01; ***p<0.001. Definition of each item provided in Table 2.

In the analysis of the three studies NCT00839423, NCT00635219 and NCT00735709, a statistically significant improvement with vortioxetine (5 and 10 mg) versus placebo was observed in change from baseline on all three insomnia items of the HAM-D (insomnia early, middle and late), two somatic items (gastrointestinal (5 mg only) and general), anxiety somatic and genital symptoms (Table 3, Figure 2). For physical symptoms measured by the HAM-A scale, a significant improvement with vortioxetine versus placebo was observed on the somatic muscular, respiratory, and gastrointestinal items for 5 mg vortioxetine, genitourinary item (5 and 10 mg vortioxetine) and autonomic item (10 mg vortioxetine) (Table 4, Figure 3). A borderline significant effect was also observed on the somatic sensory item (*p*=0.05). In the subgroup of MDD patients with a high baseline level of anxiety, significant favourable effects versus placebo for both 5 and 10 mg vortioxetine were observed on the HAM-D items of early and middle insomnia, general somatic and somatic anxiety symptoms, and genital symptoms (Table 5).

## Discussion

In this meta-analysis of five short-term randomized clinical trials in patients with MDD, vortioxetine significantly improved most of the physical symptoms of depression, as measured by the HAM-D and HAM-A. Further, in the subset of MDD patients with high baseline anxiety levels, improvements in physical symptoms were also observed.

The presence of physical symptoms in MDD patients is a significant predictor of a more chronic course of disease, with a lower probability of treatment response and remission of depressive symptoms (Gerrits et al., 2012; Jaracz et al., 2016). Residual physical symptoms may also increase the risk of recurrence (Greden, 2003).

Since comorbid anxiety disorders are observed in a substantial proportion of MDD patients (with ranges of ~30–50% reported depending upon population sampled; Kessler et al., 2015), the efficacy of vortioxetine on the physical symptoms of depression in MDD patients with marked coexisting anxiety symptoms is encouraging. A recent meta-analysis of 10 short-term randomized, placebo-controlled trials of vortioxetine in MDD patients with high levels of anxiety indicated efficacy in reducing depressive and anxiety symptoms in this group of patients (Baldwin et al., 2016b). Together with the results reported here on the physical symptoms of depression, vortioxetine also seems to be a rational treatment option in patients with MDD and high anxiety, who often do not respond satisfactorily to alternative antidepressant therapy.

Although much remains uncertain about the pathophysiology of depression, abnormalities in serotonin (5-HT) and norepinephrine (NE) neurotransmission are probably involved in psychological and physical depressive symptoms (Fava, 2003). Pain control, for instance, appears to be influenced by both 5-HT and NE; this is consistent with reports that their analgesic effects seem to be mediated via common descending pain pathways (Fava, 2003; Jones, 1991; Richardson, 1990; Willis and Westlund, 1997). The 5-HT_7_ receptor has been shown in preclinical studies to play a key role in regulation of circadian rhythmicity and sleep – physiological functions that often are disturbed in patients with MDD (Hedlund, 2009; Monti and Jantos, 2014). Non-clinical studies with vortioxetine have shown that the compound modulates several neurotransmitter systems, including GABAergic, glutamatergic, serotonergic, norepinephrinergic, dopaminergic, histaminergic and cholinergic systems through complex mechanisms involving SERT inhibition and modulation of several 5-HT receptor subtypes, including the 5-HT_7_ receptor (Sanchez et al., 2015). Further, in rodent preclinical models of analgesic activity, vortioxetine showed potential for mitigating centrally mediated pain, though no activity was observed against inflammatory pain (Committee for Medicinal Products for Human Use (CHMP), 2013). Modulation of neurotransmitters involved in neural pain pathways may mediate an analgesic response and consequently relief of painful physical symptoms associated with depression (Kelliny et al., 2015; Kurian et al., 2009; Mork et al., 2012).

Sexual dysfunction is a common physical symptom of depression as well as common side effect of many antidepressants. In the clinical development programme of vortioxetine, treatment-emergent sexual dysfunction (TESD) was prospectively captured by the Arizona Sexual Dysfunction Scale and compared with placebo. Vortioxetine 5–20 mg was associated with an approximately 5% increase in incidence of TESD, a relatively low level compared with other antidepressants (Jacobsen et al., 2016; Kennedy et al., 2016). In a recent randomized, double-blind trial in which well-treated MDD patients experiencing selective serotonin reuptake inhibitor (SSRI)-related sexual dysfunction were switched to either vortioxetine or escitalopram, significant clinical improvements in sexual functioning were observed for vortioxetine versus escitalopram, thus confirming its clinical value for this specific yet important physical symptom of depression (Jacobsen et al., 2015b).

Antidepressants with proven efficacy across multiple symptom domains may provide clinicians with important options to fill the existing unmet needs in the treatment of MDD. Vortioxetine has proven effective across a broad range of depressive symptoms as measured by MADRS or the HAM-D (Kelliny et al., 2015). In addition, vortioxetine significantly improves cognitive symptoms known to be impacted in MDD such as executive function, attention/speed of processing and memory (Harrison et al., 2016; McIntyre et al., 2016) as well as functional capacity (Christensen et al., 2018; Mahableshwarkar et al., 2015). These improvements, along with beneficial effects on physical symptoms, may confer the MDD patient the best chance for a full functional recovery.

There are some limitations to these analyses that affect the interpretation of data. All analyses were conducted post hoc using data from five short-term studies originally designed to assess a different primary outcome. In these studies, the assessment of somatic symptoms was not a specific endpoint, nor was a specific scale used for the evaluation of somatic symptoms such as measures of pain or insomnia. Nevertheless, among the commonly used scales for measuring broad antidepressant effect in clinical registration trials such as the MADRS or HAM-D, the HAM-D captures the most physical symptoms of depression in a broad MDD population. Additionally, vortioxetine is an approved antidepressant in the dose range of 5, 10, 15 and 20 mg. The HAM-D scale was only used as a measure of antidepressant effect in the studies investigating the efficacy of vortioxetine 5 and 10 mg, and therefore this study could not investigate the efficacy on physical symptoms of depression at the doses 15 and 20 mg. Nevertheless, the clinical development programme for vortioxetine demonstrated a dose–response relationship for overall efficacy, and single-item analysis of the MADRS scale confirmed this dose–response relationship across the dose range (Thase et al., 2016). Finally, our analysis is based on studies of short duration and study participants are not necessarily representative of patients with MDD in usual clinical practice.

In conclusion, the findings of these analyses indicate that patients with MDD (and patients with MDD and a high level of anxiety symptoms) can have significant improvements in MDD-associated physical symptoms during vortioxetine treatment. These findings are important in the treatment of MDD patients for the therapeutic goals of providing broad symptom relief and achieving full functional recovery.
